# Is the mental health of couples with twins more at risk? Results from an Italian cohort study

**DOI:** 10.3389/fpsyt.2024.1284090

**Published:** 2024-01-29

**Authors:** Giulia Bonanni, Valentina Laurita Longo, Chiara Airoldi, Federica Meli, Alessandra Familiari, Federica Romanzi, Marcella Pellegrino, Daniela Visconti, Annamaria Serio, Antonio Lanzone, Elisa Bevilacqua

**Affiliations:** ^1^ Unit of Obstetrics and Gynecology, Catholic University of the Sacred Heart, Rome, Italy; ^2^ Department of Women and Child Health, Women Health Area, Fondazione Policlinico Universitario Agostino Gemelli IRCCS, Rome, Italy; ^3^ Department of Translation Medicine, University of Piemonte Orientale, Novara, Italy; ^4^ Department of Clinical Psychology, Catholic University of the Sacred Heart, Rome, Italy

**Keywords:** twin pregnancies, multiples, twins, postpartum depression, maternal mental health, postnatal psychological distress, parenting stress

## Abstract

**Introduction:**

Our retrospective study aimed to investigate whether parents of twins encounter heightened psychological and emotional distress one year after childbirth, in comparison to parents of singletons within an Italian cohort.

**Methods:**

Exclusion criteria included multiparity, preterm birth, congenital anomalies, stillbirth, >2 fetus pregnancies, and pre-existing maternal mental health disorders. Out of the 300 couples (600 parents) invited to participate, 286 parents (158 mothers, 128 fathers) successfully completed a self-administered survey. We analyzed three scores separately for mothers and fathers, differentiating between singleton and twin pregnancies: the Edinburgh Postnatal Depression Scale (EPDS) score, the State and Trait Anxiety Inventory (STAI)-Y1 score, and the STAI-Y2 score.

**Results:**

Logistic models were used to assess the influence of age, BMI, marital status, education, and employment on the three binary scores (EPDS, STAI-Y1, and STAI-Y2), revealing no significant differences in absolute scores between parents of singletons and twins. Paired analysis revealed significantly higher EPDS (mean increase: 3.8, SD: 6.5), STAI-Y1 (mean increase: 5.4, SD: 12.5), and STAI-Y2 (mean increase: 4.5, SD: 12.4) scores for mothers (p < 0.0001). Approximately 10% of women and 8% of men reported suicidal thoughts.

**Discussion:**

Contrary to expectations, no substantial psychological differences emerged between parents of twins and singletons. Adjusting for confounders through univariate analysis maintained nonsignificant trends. Nevertheless, caution in interpretation is warranted due to strict inclusion criteria favoring twin pregnancies with better outcomes. Unintended bias could have resulted from routine psychological support offered to mothers of twins in our clinic. This presents an important framework for future research, including randomized controlled trials comparing parents of multiples with psychological support to those without.Finally, the elevated prevalence of depression symptoms and suicidal thoughts in our cohort underscores the importance of mental health during pregnancy and early parenting. We advocate for the screening of parents for postpartum depression and various psychological conditions, encompassing a spectrum of anxiety disorders. Those at elevated risk of mental distress should be proactively offered appropriate support.

## 1 Introduction

The last decades have witnessed a significant increase in multiple pregnancy rates (1). The widespread of Assisted Reproduction Techniques (ART) and the rising maternal age at conception have been the major contributors to a roughly 70% increase in these pregnancies over the last fifty years (2). The heightened incidence of adverse outcomes, such as stillbirths (3, 4), neonatal deaths (5), and cerebral palsy (6), has fueled extensive research and clinical interest in multiple pregnancies. Notably, a significant proportion of twins (25%), triplets (75%), and quadruplets (100%) necessitate admission to a neonatal intensive care unit (7). While existing studies predominantly focus on neonatal and maternal risks linked with multiple pregnancies, the realm of perinatal mental health in parents of twins remains an underexplored facet in contemporary research. Recognizing this gap, the Global Twins and Multiples Priority Setting Partnership recently identified it as a top priority in the health research landscape for multiples (8).

Understanding the prevalence of anxiety and depression during the antenatal and postnatal periods is crucial for addressing the complexities of perinatal mental health. In a comprehensive 2017 meta-analysis, Falah-Hassani et al. (9) synthesized data from 66 studies encompassing 162,120 women across 30 countries. The findings disclosed a prevalence of 9.5% for self-reported antenatal anxiety symptoms and mild to severe depressive symptoms. Moreover, co-morbid anxiety symptoms alongside moderate to severe depressive symptoms were observed in 6.3% of cases. Concerning fathers, the prevalence of paternal postpartum depression was reported to be around 4%–25% globally (10). A recent systematic review and meta-analysis by Smythe et al. (11) delved into 23 studies involving 29,286 couples. The investigation revealed that up to 3.18% of parental dyads, comprising both mothers and fathers, experienced perinatal depression. Notably, the prevalence was higher during the late postnatal period (3-12 months) (11).

When families receive the news of expecting multiple pregnancies, it becomes imperative to consider their mental well-being alongside conventional concerns. Beyond the typical challenges tied to pregnancy and newborn care, parents of multiples face added stress like increased caregiving demands, lack of sleep, financial strain, and social isolation, leading to higher risks of depression and anxiety (12). Recognizing and addressing the mental health of parents of twins is essential. Existing research that compares the mental health of parents with multiples to those with singletons has yielded mixed results, partly attributable to variations in outcome measurement. A study by Choi et al. (13) revealed that mothers of multiples had a 43% higher likelihood of experiencing moderate/severe depressive symptoms at 9 months postpartum compared to mothers of singletons (13). The first 3 months of postpartum are widely recognized as the most challenging for parents of multiples (14), marked by stress, overwhelm, and sleep deprivation (15). However, our clinical experience during follow-ups led us to hypothesize that parents of multiples face unique stressors extending beyond the initial postpartum phase, including ongoing sleep deprivation, financial burdens, and social isolation related to pregnancy-related factors.

The primary aim of our study was to establish whether parents of twins demonstrate a heightened likelihood of experiencing psychological and emotional distress, specifically anxiety and depression, one year after birth, in comparison to parents of singletons within an Italian cohort. Additionally, as a secondary aim, we aimed to assess the difference between mothers and fathers within the couple in terms of psychological and emotional distress. Furthermore, we analyzed the association between elevated probabilities of depression and anxiety and potential sociodemographic and obstetric risk factors (16), including maternal age, Body Mass Index (BMI), educational level, and marital status.

## 2 Materials and methods

### 2.1 Study design and data collection

A retrospective cohort study was performed at the Department of Women and Child Health, Fondazione Policlinico Universitario Agostino Gemelli, IRCCS, Rome, Italy, a tertiary university hospital with an annual delivery rate of approximately 4000. Inclusion criteria were primigravid women, maternal age > 18 years, and gestational age at delivery > 36 weeks (in case of twins) or > 37 weeks (in case of singletons). Exclusion criteria included congenital anomalies, stillbirth, multiple pregnancies with more than two fetuses, and maternal pre-existing mental health disorders. Over a 12-month period (November 2018 to November 2019), we randomly selected three hundred couples who delivered at our institution. The appropriate sample size was established by first conducting a comprehensive literature review to identify analogous studies, leveraging their sample sizes as an initial reference. Subsequently, we approximated the sample size required for a 95% confidence interval, employing a standard Z-score of 1.96, which aligns with a 5% margin of error. Employing a simple random sampling method based on unique patient identifiers routinely assigned by the hospital, we enrolled 300 participants. The study adhered to the principles of the Declaration of Helsinki and was approved by the Ethics Committee of Catholic University of the Sacred Heart (protocol code DIPUSVSP-16-11-2091).

The original population was balanced between singleton (n=150) and twin pregnancies (n=150). All women included in the study were contacted by phone at 1 year postpartum to be informed about the purpose and the characteristics of the study. During phone calls, women who accepted to participate and agreed to the informed consent received an email containing a link to the questionnaire. The same link was also sent to their partners. Socio-demographic and obstetric data (including maternal age, BMI, nationality, educational level, employment, and marital status) were extracted from a pre-existing perinatal database where data from all women who delivered at our Institution are prospectively included; then, we codified these data on an electronic file. All records were reviewed for the purpose of the study by a single reviewer (VLL). An Excel file was created matching pre-existing information (including maternal age, BMI, nationality, educational level, employment, and marital status) with results obtained from three questionnaires (**Supplementary Materials**): the EDPS (Edinburgh Postnatal Depression Scale), STAI-Y1 (State and Trait Anxiety Inventory), and STAI-Y2 scales.

### 2.2 Definition of variables and outcomes

Psychological and emotional distress were evaluated utilizing a pair of self-report questionnaires. The questionnaires yielded three distinct scores that served as the primary outcome measures: the EPDS score (17), the STAI-Y1 score, and the STAI-Y2 score (18).

#### 2.1.1 EPDS – Edinburgh Postnatal Depression Scale

The EPDS, a widely utilized tool internationally, is employed for the screening of Post-Partum Depression (PPD), aiming to identify the onset of major depressive disorder within one year after childbirth. Comprising 10 questions, each statement presents four potential responses, scored from 0 to 3. Higher scores indicate more pronounced depressive symptoms, with a maximum score of 30. We categorized scores into four groups: Group I (≤ 8), suggesting unlikely depression; Group II (9-11), indicative of possible depression; Group III (12-13), signaling a fairly high likelihood of depression; and Group IV (≥ 14), signifying probable depression.

In addition, we conducted a separate analysis considering a positive score on question 10 (“The thought of harming myself has occurred”) as a secondary outcome, identifying individuals with potential suicidal risk (17, 19). Further, we dichotomized scores using a cutoff of 11 or higher, implying the possibility of PPD warranting additional clinical investigation, such as a psychological assessment or an interview with a social worker. According to a meta-analysis on pregnant and postpartum women by Levis et al. (20), the EPDS cutoff value of 11 or higher maximizes combined sensitivity and specificity in women (20). Regarding the suggested cutoff for fathers, a recent systematic review and meta-analysis validated EPDS for detecting paternal postpartum depression, with lower cutoff scores ranging from 7 to 10 (21).

#### 2.1.2 STAI – State and Trait Anxiety Inventory

The State-Trait Anxiety Inventory (STAI) is a widely utilized tool for measuring trait and state anxiety, with clinical applications in diagnosing anxiety disorders and distinguishing between anxiety and depressive syndromes. The STAI most popular version (Y) consists of 20 items assessing trait anxiety and 20 items assessing state anxiety. State anxiety items include descriptors such as “I am tense; I am worried” and “I feel calm; I feel secure,” while trait anxiety items encompass statements like “I worry too much over something that really doesn’t matter” and “I am content; I am a steady person.” Responses are rated on a 4-point scale (e.g., from “Almost Never” to “Almost Always”), with higher scores indicating an elevated risk of anxiety (18). Scores for version Y of the STAI range from a minimum of 20 to a maximum of 80. STAI scores are often classified as follows: “no or low anxiety” (20-37), “moderate anxiety” (38-44), and “high anxiety” (45-80) (22). Alternatively, scores of 42 or higher (for STAI-Y1) and 43 or higher (for STAI-Y2) have been proposed to detect clinically significant symptoms and differentiate between healthy adults and those with anxiety disorders (18, 23). The internal consistency coefficient was previously reported as the same for mothers and fathers (24).

### 2.3 Statistical analysis

A comprehensive descriptive statistical analysis was conducted on both the entire sample and distinctively for singleton and twin pregnancies. For categorical variables, absolute and relative frequencies were presented, while numerical variables were summarized using either means and standard deviations or medians and interquartile ranges, as appropriate. The correlation between psychological scores was evaluated by calculating the Pearson correlation coefficient and presenting the scatter plots. In this manner, no distinction was made between mothers and fathers.

In order to discern disparities in psychological and emotional distress, a comparative analysis of psychological scores was undertaken between the two groups (singleton vs. twin pregnancies), independently for mothers and fathers. These scores were evaluated in both categorical and continuous manners. The applicable statistical tests, namely Chi-square/Fisher test and t-test/Mann-Whitney, were employed for these comparisons. Additionally, paired analysis tests were conducted to assess score differences between mothers and fathers within the same family.

Furthermore, the influence of potential risk factors on psychological and emotional distress was examined using logistic univariate models. Three such models were estimated, utilizing dichotomous scores (EPDS, STAI-Y1, and STAI-Y2) as outcomes and incorporating all available covariates (age, BMI, marital status, education, employment) as controls. Adjustment was made for singleton and twin pregnancies, and odds ratios with corresponding 95% confidence intervals were provided.

To explore the interrelation between psychological scores, the Pearson correlation coefficient was computed, accompanied by scatter plots to visually represent the relationships.

No correction for multiple comparisons was undertaken as all analyses were pre-specified in the protocol. The significance threshold was set at 0.05 (two-tailed), and all analyses were executed using SAS 9.4 software.

## 3 Results

**Figure 1:**
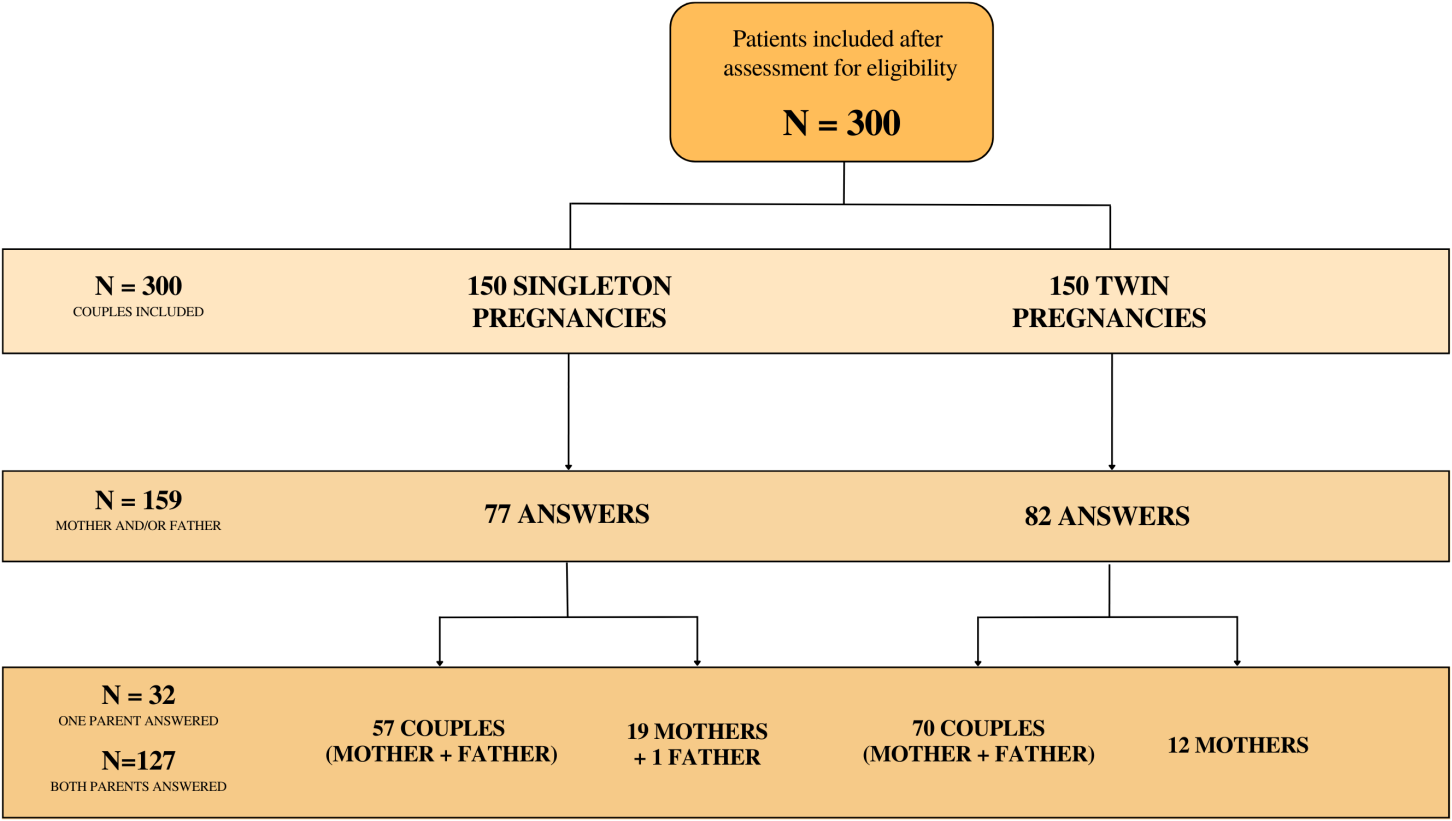
Design and flow of participants through the study.

Three hundred couples were invited to participate (**Figure 1**). Out of these, 159 (53.00%) provided informed consent and responded to the questionnaire. The responses included those from only the mother (n=31), only the father (n=1), or both the mother and the father (n=127). The response rate was consistent between singleton and twin pregnancies, at 77 (51.33%) and 82 (54.67%) respectively. In total, the number of mothers who responded to the questionnaire was 158 (57 + 19 + 70 + 12), while the number of fathers was 128 (57 + 1 + 70).

The baseline sociodemographic characteristics of the entire cohort are summarized in **Table 1**, both for singletons and twins. All responding parents were different-sex couples. The mean maternal age at the time of delivery was 34.0 years (SD 4.9), ranging from 21 to 48 years. The median maternal weight and BMI at delivery were 62.0 kg [IQR 56.0-70.0] and 22.8 kg/m2 [IQR 20.5-25.7], respectively. White ethnicity was the most prevalent (n=154, 97.5%). Approximately 80% (n=142) of the women were married, and around 73.1% (n=106) were employed during pregnancy. Nearly all women who participated in the study had received secondary education or higher (147; 96.1%), and they delivered using the NHS (132; 95.7%).

**Table 1:** Baseline characteristics of both the maternal and paternal cohorts are presented, along with separate analyses for singleton and twin pregnancies.

*Characteristic*	*Mother*		
	*All (n=158)*	*Singleton (n=76)*	*Twin (n=82)*
***Age*** *, years, mean (SD)*	33.99 (4.85)	33.84 (4.23)	34.13 (5.39)
***Height*** *, cm, mean (SD)*	166.36 (5.35)	166.33 (5.86)	166.39 (5.23)
***Weight*,** *kg, median [IQR]*	62.00 [56.00; 70.00]	60.00 [56.00; 67.50]	65.00 [56.00; 76.00]
***Post-partum BMI*** *, kg/m^2^, median [IQR]*	22.83 [20.52; 25.71]	21.84 [20.18; 24.42]	24.02 [20.70; 26.99]
***White*** *, n (%)*	154 (97.47)	74 (97.37)	80 (97.56)
***Italian*** *, n (%)*	142 (89.87)	69 (90.79)	73 (89.02)
***Married*** *, n (%)*	123 (80.39)	55 (77.46)	68 (82.93)
***Secondary or higher education*** *, n (%)*	147 (96.08)	70 (98.59)	77 (93.90)
***Employed*** *, n (%)*	106 (73.10)	51 (80.95)	55 (67.07)
***NHS delivery*** *, n (%)*	132 (95.65)	73 (9.33)	59 (93.65)
	*Father*		
	*All (n=128)*	*Singleton (n=58)*	*Twin (n=70)*
***Age*** *, years, mean (SD)*	37.05 (5.78)	36.55 (4.94)	37.45 (6.39)
***Height*** *, cm, mean (SD)*	177.95 (6.35)	177.81 (6.36)	178.06 (6.39)
***Weight*,** *kg, median [IQR]*	80.00 [72.00; 87.00]	80.00 [72.00; 86.00]	79.00 [72.00; 88.00]
***Post-partum BMI*** *, kg/m^2^, median [IQR]*	24.84 [23.15; 27.44]	25.40 [23.18; 27.70]	24.69 [23.15; 27.14]
***White*** *, n (%)*	126 (97.67)	55 (94.83)	71 (100.00)
***Italian*** *, n (%), missing=50*	75 (94.94)	8 (100.00)	67 (94.37)
***Married*** *, n (%)*	126 (97.67)	58 (100.00)	68 (95.77)
***Secondary or higher education*** *, n (%)*	113 (87.60)	56 (96.55)	57 (80.28)
***Employed*** *, n (%)*	113 (88.28)	46 (79.31)	67 (95.71)

Categorical variables are expressed as absolute and relative frequencies, while numerical variables are represented using means and standard deviations (SD), or medians and interquartile ranges (IQR) where applicable. BMI, Body Mass Index; NHS, National Health System.

The men included in the analysis were slightly older, with a mean father age at delivery of 37.1 years (SD 5.8). Almost all fathers were of white ethnicity (n=126; 97.7%). Most men participating in the study were married (n=126; 97.7%), stably employed (n=113; 88.3%), and had received a secondary education or higher (n=113; 87.6%). As shown in **Table 1**, no significant differences were observed between singletons and twins, except for post-partum weight (0.007) and BMI (0.006) in mothers. As expected, these measures were higher in mothers with twin pregnancies compared to mothers of singletons.

Psychological and emotional distress were assessed using EPDS, STAI-Y1, and STAI-Y2 scores. A high positive correlation (r^2^ > 0.700) was observed among these scores, indicating a strong association between post-partum depression and state and trait anxiety. Further details are provided in **Supplementary Figure 1**.

**Figure 2:**
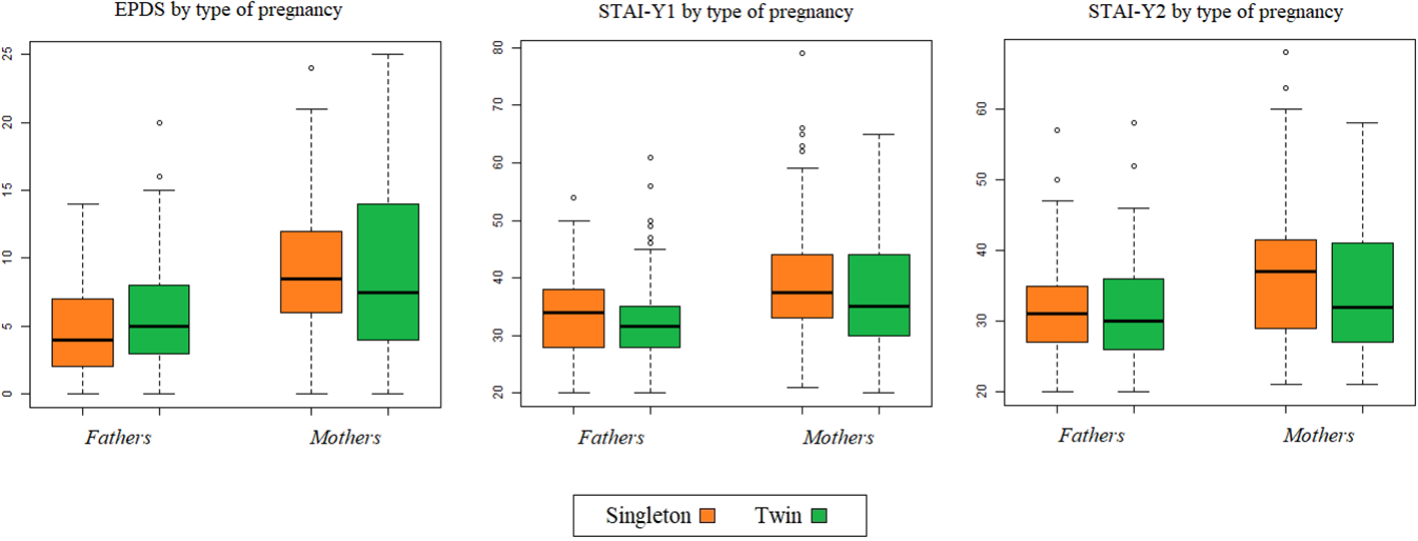
Boxplot of type of pregnancy and psychological scores, separated for fathers and mothers.

The psychological scores collected are summarized in **Table 2** and visualized in **Figure 2**. Overall, 36 (22.8%) women and 5 (3.9%) men exhibited a higher likelihood of depression based on EPDS scores, with 15 (9.5%) mothers and 10 (7.8%) fathers showing signs of suicidal risk. The mean EPDS score was 9.1 (SD 5.7) for women and 5.4 (SD 3.9) for men. Among the sampled participants, 24.0% of mothers experienced trait anxiety, and 19.6% experienced state anxiety, compared to 10.2% and 6.3%, respectively, among fathers. Specifically, the mean STAI-Y1 and STAI-Y2 scores exceeded 35 for women and 31 for men. No statistically significant differences were observed in EPDS and STAI-Y1/2 scores between parents of singletons and twins, regardless of whether the variables were considered categorically or continuously. Upon closer examination of categorized scores, a slight, albeit nonsignificant, higher prevalence of depressive symptoms and a greater probability of trait and state anxiety were observed in the twin groups compared to singletons, except for STAI-Y2 in women. Approximately 10% of women and 8% of men exhibited suspected suicidal thoughts; although risk appeared somewhat higher among mothers of twins at the 10% level (p= 0.07), this difference did not reach statistical significance at the 5% level.

**Table 2:** EPDS, STAI-Y1, and STAI-Y2 scores by parent and pregnancy type.

	*Mothers*			
	All (n=158)	Singleton (n=76)	Twin (n=82)	*p-value*
*EPDS*				
*Suicidal risk, n (%)*	15 (9.49)	4 (5.19)	11 (13.58)	0.3073
*EPDS*				
*Depression not likely, n (%)*	81 (51.27)	38 (50.00)	43 (52.44)	0.3685
*Depression possible, n (%)*	30 (18.99)	18 (23.68)	12 (14.63)	
*Fairly high possibility of depression, n (%)*	11 (6.96)	6 (7.89)	5 (6.10)	
*Higher probable depression, n (%)*	36 (22.78)	14 (18.42)	22 (26.83)	
***EPDS total*** *, Mean (SD)*	9.09 (5.70)	9.14 (4.85)	9.05 (6.41)	0.9161
*STAI-Y1*				
*No or low anxiety, n (%)*	86 (54.43)	38 (50.00)	48 (58.54)	0.3496
*Moderate anxiety, n (%)*	34 (21.52)	20 (26.32)	14 (17.07)	
*High anxiety, n (%)*	38 (24.05)	18 (23.68)	20 (24.39)	
***STAI-Y1 total*,** *Mean (SD)*	38.34 (10.26)	39.62 (10.60)	37.15 (9.85)	0.1307
*STAI-Y2*				
*No or low anxiety, n (%)*	95 (60.13)	42 (55.26)	53 (64.63)	0.4538
*Moderate anxiety, n (%)*	32 (20.25)	18 (23.68)	14 (17.07)	
*High anxiety, n (%)*	31 (19.62)	16 (21.05)	15 (18.29)	
***STAI-Y2 total*,** *Mean (SD)*	35.97 (9.94)	37.17 (9.93)	34.87 (9.89)	0.1460
	*Fathers*			
	All (n=128)	Singleton (n=58)	Twin (n=70)	*p-value*
*EPDS*				
*Suicidal risk, n (%)*	10 (7.81)	5 (7.14)	5 (8.62)	0.2664
*EPDS*				
*Depression not likely, n (%)*	103 (80.47)	50 (86.21)	53 (75.71)	0.4257
*Depression possible, n (%)*	16 (12.50)	6 (10.34)	10 (14.29)	
*Fairly high possibility of depression, n (%)*	4 (3.13)	1 (1.72)	3 (4.29)	
*Higher probable depression, n (%)*	5 (3.91)	1 (1.72)	4 (5.71)	
***EPDS total*** *, Mean (SD)*	5.41 (3.88)	4.72 (3.44)	5.97 (4.15)	0.0700
*STAI-Y1*				
*No or low anxiety, n (%)*	96 (75.00)	43 (74.14)	53 (75.71)	0.7162
*Moderate anxiety, n (%)*	19 (14.84)	10 (17.24)	9 (12.86)	
*High anxiety, n (%)*	13 (10.16)	5 (8.62)	8 (11.43)	
***STAI-Y1 total*,** *Mean (SD)*	33.41 (7.77)	33.79 (7.17)	33.09 (8.27)	0.6101
*STAI-Y2*				
*No or low anxiety, n (%)*	104 (81.25)	48 (82.76)	56 (80.00)	0.8858
*Moderate anxiety, n (%)*	16 (12.50)	7 (12.07)	9 (12.86)	
*High anxiety, n (%)*	8 (6.25)	3 (5.17)	5 (7.14)	
***STAI-Y2 total*,** *Mean (SD)*	31.95 (7.57)	32.00 (7.26)	31.90 (7.86)	0.9410

Scoring categories for EPDS, STAI-Y1, and STAI-Y2 are presented, separately for mothers and fathers, based on singleton and twin pregnancies. Absolute/relative frequencies and means/SDs, along with p-values from Chi-square/Fisher tests (categorical) and t-student tests (numerical) are included for inter-group comparisons. EPDS, Edinburgh Postnatal Depression Scale; STAI, State and Trait Anxiety Inventory.

When focusing solely on pregnancies with both partners participating (n=127), paired comparisons of scores between mothers and fathers were conducted. Mothers exhibited significantly higher scores with an increase of 3.8 (SD 6.5) for EPDS; 5.4 (SD 12.5) for STAI-Y1 and 4.5 (SD 12.4) for STAI-Y2. These differences were statistically different from 0 (p<0.0001). These discrepancies were slightly more pronounced in singleton pregnancies, both in continuous and categorical ways. More details are reported in **Supplementary Table 1**.

Subsequently, we conducted a series of univariate and multivariable logistic regression models, employing higher EPDS (≥11), STAI-Y1 (≥42), and STAI-Y2 (≥43) scores in a dichotomous way as the focal outcomes. Particularly, high-risk depression was observed for 52 (32.9%) women and 13 (10.2%) men, a high level of trait anxiety for 49 (31.0%) mothers and 21 (16.4%) fathers and a high level of state anxiety for 36 (22.8%) and 13 (10.2%), respectively. More details on this categorization are reported in **Supplementary Table 2**. None of the univariate analyses yielded statistically significant findings, thus emphasizing the absence of conclusive associations.

Importantly, upon comprehensive adjustment for potential confounders (including age, BMI, marital status, education, and employment) through univariate analysis, the overall direction and the statistical significance of the results remained consistently non-significant except for mother civil status on STAI-Y1 and father age in EPDS: married women had lower risk of suffered from state anxiety [OR 0.38, 95% CI 0.17; 0.87] and increasing the father ages decrease the risk of depression [OR 0.89; 95% CI 0.79; 0.99]. No significant differences were observed among twins and singletons (**Table 3**).

**Table 3:** Univariate logistic regression models, employing higher EPDS (≥11), STAI-Y1 (≥42), and STAI-Y2 (≥43) scores in a dichotomous way as the focal outcomes.

*Mother*	EPDS	STAI-Y1	STAI-Y2
	*OR [95% CI]*	*OR [95% CI]*	*OR [95% CI]*
*Twin vs singleton*	1.42 [0.73; 2.77]	0.95 [0.48; 1.87]	0.91 [0.43; 1.91]
*Age, years*	1.01 [0.94; 1.08]	1.00 [0.93; 1.07]	0.97 [0.90; 1.05]
*BMI, kg/m^2^*	1.05 [0.97; 1.13]	1.02 [0.94; 1.10]	1.04 [0.95; 1.13]
*Married vs single*	0.51 [0.23; 1.15]	**0.38 [0.17; 0.87]**	0.54 [0.22; 1.28]
*Secondary or higher vs low education*	0.50 [0.10; 2.57]	0.91 [0.16; 5.15]	0.56 [0.10; 3.18]
*Employed vs unemployed*	1.03 [0.47; 2.24]	1.02 [0.46; 2.25]	0.63 [0.27; 1.46]
*Father*	EPDS	STAI-Y1	STAI-Y2
	*OR [95% CI]*	*OR [95% CI]*	*OR [95% CI]*
*Twin vs singleton*	1.99 [0.58; 6.84]	1.43 [0.55; 3.72]	1.37 [0.42; 4.43]
*Age, years*	**0.89 [0.79; 0.99]**	0.94 [0.86; 1.03]	0.90 [0.81; 1.01]
*BMI, kg/m^2^*	1.00 [0.87; 1.15]	**0.83 [0.70; 0.98]**	0.94 [0.80; 1.11]
*Married vs single*	–	–	–
*Secondary or higher vs low education*	0.76 [0.15; 3.80]	0.83 [0.21; 3.21]	0.76 [0.15; 3.80]
*Employed vs unemployed*	–	3.01 [0.37; 24.21]	1.66 [0.20; 13.79]

Bolded values indicate confidence intervals that do not include 1, indicating statistical significance.-, missing values for some variables are due to instability in the estimation of odds ratios and confidence intervals, making them unavailable or unreliable.

## 4 Discussion

In this study, contrary to our initial expectations, we did not observe a significant difference in psychological and emotional distress between parents of twins and parents of singletons. However, when examining partners within the study cohort, mothers consistently displayed significantly higher median scores for Postpartum Depression (PPD), trait anxiety, and state anxiety compared to fathers (all p<0.001). Notably, this trend was more pronounced in singleton pregnancies. Despite thorough adjustments for potential confounders, including age, BMI, marital status, education, and employment, the inherent association between twin pregnancies and elevated EPDS scores in both partners persisted.

The greater disparity in mental health symptoms between mothers of singletons and their partners compared to mothers of twins and their partners could be attributed to different underlying factors. The heightened level of paternal involvement in parenting among fathers of twins (25, 26), as opposed to fathers of singletons, might contribute to a more balanced distribution of parenting-related stressors and responsibilities. This increased paternal involvement could potentially lead to fathers of twins experiencing mental health outcomes more closely resembling those of mothers. Additionally, other factors may come into play, including the unique challenges faced by parents of twins, fostering a shared understanding, and potentially cultivating greater mutual support between partners. Future investigations on this topic are warranted to delve deeper into these dynamics and provide a more comprehensive understanding of the implications for parental mental health.

In the context of existing literature comparing parents of twins to parents of singletons, our findings contrast with specific earlier studies (13, 27–32), yet align with the consistency demonstrated in numerous other investigations (33–39). The observed variability in scientific literature could be attributed to various factors. Primarily, the presence of high levels of heterogeneity in outcome measures assessing the risk of depression and anxiety often poses challenges in making comparisons and achieving result standardization. For instance, studies that failed to identify inter-group disparities in PPD predominantly utilized the EPDS (12). Furthermore, discrepancies can arise from differences in inclusion criteria across these studies. Notably, our research benefits from a robust framework with well-defined inclusion criteria, rigorously excluding instances of preterm births, fetal anomalies, and severe maternal comorbidities. However, it is important to acknowledge a limitation of our study, namely the absence of *In Vitro* Fertilization (IVF) data, which hindered our ability to control for a factor more frequently associated with a heightened risk of psychological impairment.

While this study sheds light on various dimensions, certain limitations warrant consideration. The relatively small sample size necessitates cautious interpretation of the results, as the statistical power may be limited. The low response rate of 53% may have introduced non-response bias, potentially skewing the sample toward a representation of individuals more inclined to participate due to milder distress levels. Additionally, the retrospective design of the study posed challenges in administering questionnaires and restricted participant inclusion. While excluding preterm birth or congenital anomalies allowed for a focused examination of unique psychological aspects associated with multiple pregnancies, without the confounding influence of adverse medical outcomes, we acknowledge that it may have inadvertently selected a group of twin pregnancies with better outcomes. This could indeed be a limitation, as our study population may not be fully representative of the broader spectrum of twin pregnancies, often characterized by higher medical complexities.

Our established practice at the multiple pregnancy specialty program, where we offer complimentary psychological support to mothers of twins at no cost, may have inadvertently introduced bias. The presence of a multidisciplinary staff, including a specialized nurse and a psychologist, could have influenced our results and mitigated the previously observed differences in anxiety and depression levels between mothers of twins and mothers of singletons. This distinctive approach, in contrast to the predominantly medicalized perspective on twin pregnancies observed in certain healthcare systems, likely played a pivotal role in the comparable mental health outcomes between parents of twins and parents of singletons. We highlight this care model as a potential explanation for our findings, proposing it as a noteworthy consideration for future research.

It is important to contextualize our study within the societal framework of Italy, where new parents, including those with twins, benefit from income-based economic support through the ‘Baby Bonus.’ This support increases for the second child, as is the case with twins, yet it remains a modest contribution (ranging from EUR 80/month to EUR 160/month). Maternal and paternal leave is consistent for singletons and twins, fully compensated for 5 months and partially compensated for an additional 6 months.

It is important to acknowledge the unintentional absence of same-sex couples and the predominantly white composition (98%) in our sample. The experiences and psychosocial factors of same-sex couples may differ, limiting the generalizability of our findings to these populations. Additionally, our study did not comprehensively address cultural and racial factors due to the predominantly white sample, potentially impacting the broader applicability of our results. A noteworthy aspect is that most participants were married, suggesting stronger social support and healthier relationships with their domestic partners. This, coupled with the psychological assistance provided, could have influenced our findings. These considerations provide a foundational context for future studies, underlining the need for diverse samples and exploring the impact of psychological support on parents of multiples, particularly within various cultural and relational contexts.

A notable strength of our study is the inclusion of fathers in the investigation, an area that has been understudied in the past. The higher prevalences of EPDS scores among fathers of twins compared to fathers of singletons in our cohort may signify potential disparities in support. This highlights an important avenue for future research to delve deeper into understanding the unique challenges fathers face in coping with twin pregnancies.

In conclusion, our study reveals no significant overall distress differences between parents of twins and singletons at one year postpartum, yet highlights a consistent trend of higher postpartum depression and anxiety scores among mothers when compared to fathers, particularly in singleton pregnancies. Our results also emphasize the critical need to prioritize mental health in prenatal care. When pregnancies are diagnosed, the mental health of families should be placed on an equal footing with other concerns. The observed high prevalence of depression symptoms and suicidal thoughts, notably among mothers of twins, underscores the urgency of integrating mental health screenings for both singleton and twin pregnancies. Identifying and offering targeted support for postpartum depression and anxiety are deemed crucial. Moreover, active involvement of fathers can aid couples in coping with the stress of twin pregnancies. Our routine practice at the multiple pregnancy specialty program, offering no-cost psychological assistance to mothers of twins, may have influenced results, possibly reducing the previously observed difference in anxiety and depression levels between mothers of twins and singletons. While this care model offers a potential explanation for our findings, it underscores the need for future research, including a randomized controlled trial comparing parents of multiples receiving psychological support with those who do not receive such assistance, with the aim of further elucidating the impact of integrated psychological care on the well-being of parents navigating the challenges of multiple pregnancies.

## Data availability statement

The raw data supporting the conclusions of this article will be made available by the authors, without undue reservation.

## Ethics statement

The studies involving humans were approved by Ethics Committee of Catholic University of the Sacred Heart (protocol code DIPUSVSP-16-11-2091). The studies were conducted in accordance with the local legislation and institutional requirements. The participants provided their written informed consent to participate in this study.

## Author contributions

GB: Conceptualization, Data curation, Investigation, Methodology, Project administration, Resources, Writing – original draft, Writing – review & editing. VL: Conceptualization, Investigation, Methodology, Writing – original draft, Writing – review & editing. CA: Formal analysis, Writing – original draft, Writing – review & editing. FM: Investigation, Resources, Writing – review & editing. AF: Investigation, Resources, Writing – review & editing. FR: Investigation, Resources, Writing – review & editing. MP: Investigation, Resources, Writing – review & editing. DV: Investigation, Resources, Writing – review & editing. AS: Investigation, Resources, Writing – review & editing. AL: Supervision, Writing – review & editing. EB: Conceptualization, Data curation, Investigation, Methodology, Project administration, Resources, Supervision, Writing – review & editing.
