# Supplementary material for: Is the mental health of couples with twins more at risk? Results from an Italian cohort study
Source: Front Psychiatry. 2024 Jan 29;15:1284090. doi: 10.3389/fpsyt.2024.1284090 (PMC10859489; doi:10.3389/fpsyt.2024.1284090)
Supplement: Supplementary file 1 [file DataSheet_1.docx]

**Supplementary Files**

**Figure S1.** Scatter Plots of Psychological and Emotional Distress Scores.


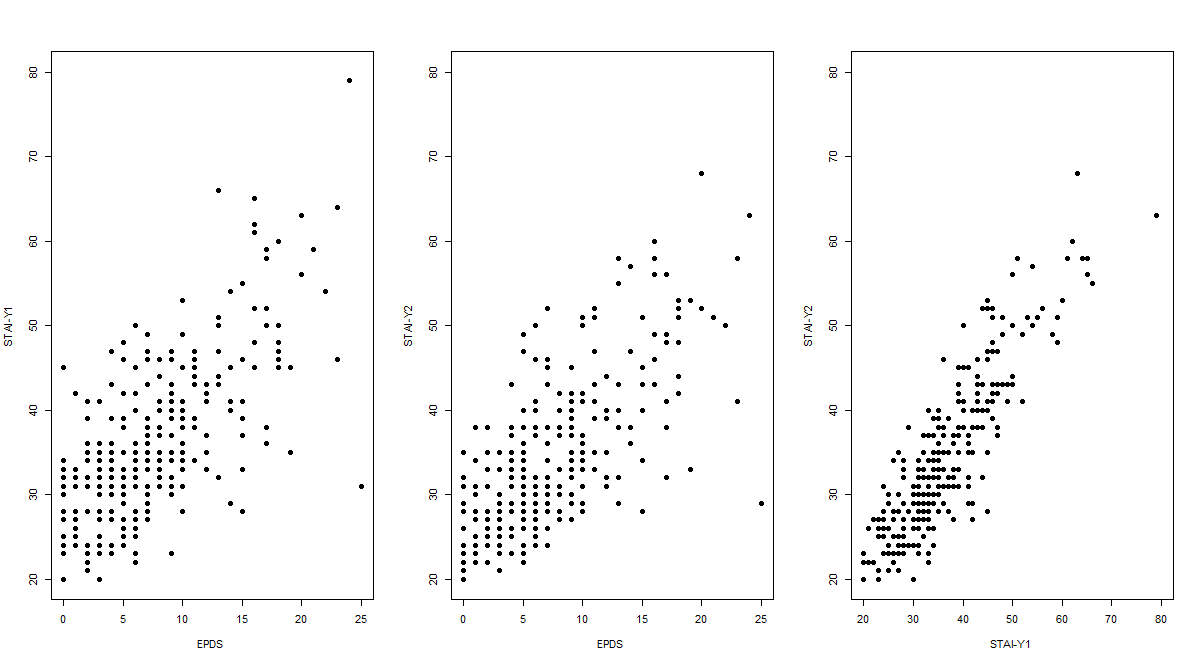


*Note: The correlations were 0.701 between EPDS and STAI-Y1, 0.709 between EPDS and STAI-Y2, and 0.872 between STAI-Y1 and STAI-Y2.*

**Table S1.** Difference of score among mother and father for couple where both partners participated in the study.

| ***Score difference (Mother vs Father)*** | | All (n=127) | | Singleton (n=57) | Twin (n=70) |
| --- | --- | --- | --- | --- | --- |
| ***EPDS*** | |  | |  |  |
| *Mother worse, n (%)* | | 29 (22.83) | | 8 (14.04) | 21 (30.00) |
| *Concordance, n (%)* | | 7 (5.51) | | 2 (3.51) | 5 (7.14) |
| *Mother better, n (%)* | | 91 (71.65) | | 47 (82.46) | 44 (62.86) |
| *Delta (mother - father), mean (SD)* | | 3.77 (6.50) | | 4.75 (5.60) | 2.97 (7.09) |
|  | |  | |  |  |
| ***STAI-Y1*** | |  | |  |  |
| *Mother worse, n (%)* | | 41 (32.28) | | 16 (28.07) | 25 (35.71) |
| *Concordance, n (%)* | | 4 (3.15) | | 2 (3.51) | 2 (2.86) |
| *Mother better, n (%)mean* | | 82 (64.57) | | 39 (68.42) | 43 (61.43) |
| *Delta (mother - father), (SD)* | | 5.43 (12.46) | | 6.96 (12.28) | 4.17 (12.55) |
|  | |  | |  |  |
| ***STAI-Y2*** | |  | |  |  |
| *Mother worse, n (%)* | | 43 (33.86) | | 16 (28.07) | 27 (38.57) |
| *Concordance, n (%)* | | 4 (3.15) | | 3 (5.26) | 1 (1.43) |
| *Mother better, n (%)* | | 80 (62.99) | | 38 (66.67) | 42 (60.00) |
| *Delta (mother - father), mean (SD)* | | 4.51 (12.44) | | 6.47 (12.66) | 2.91 (12.12) |
|  |  | |  | |  |

*Note: Means and standard deviations were reported for all, and separately for singleton and twin pregnancy. EPDS = Edinburgh Postnatal Depression Scale; STAI = State and Trait Anxiety Inventory*

**Table S2.** EPDS, STAI-Y1 and STAI-Y2 scoring categories in mother and father, separately for singleton and twin pregnancy.

| ***Mother*** | **All (n=158)** | **Singleton (n=76)** | **Twin (n=82)** |
| --- | --- | --- | --- |
|  | ***n (%)*** | ***n (%)*** | ***n (%)*** |
| ***EPDS*** |  |  |  |
| *Low (<11)* | 106 (67.09) | 54 (71.05) | 52 (63.41) |
| *High (11+)* | 52 (32.91) | 22 (28.95) | 30 (36.59) |
| ***STAI-Y1*** |  |  |  |
| *Low (<42)* | 109 (68.99) | 52 (68.42) | 57 (69.51) |
| *High (42+)* | 49 (31.01) | 24 (31.58) | 25 (30.49) |
| ***STAI-Y2*** |  |  |  |
| *Low (<43)* | 122 (77.22) | 58 (76.32) | 64 (78.05) |
| *High (43+)* | 36 (22.78) | 18 (23.68) | 18 (21.95) |
|  |  |  |  |
| ***Father*** | **All (n=128)** | **Singleton (n=58)** | **Twin (n=70)** |
|  | ***n (%)*** | ***n (%)*** | ***n (%)*** |
| ***EPDS*** |  |  |  |
| *Low (<11)* | 115 (89.84) | 54 (93.1) | 61 (87.14) |
| *High (11+)* | 13 (10.16) | 4 (6.9) | 9 (12.86) |
| ***STAI-Y1*** |  |  |  |
| *Low (<42)* | 107 (83.59) | 50 (86.21) | 57 (81.43) |
| *High (42+)* | 21 (16.41) | 8 (13.79) | 13 (18.57) |
| ***STAI-Y2*** |  |  |  |
| *Low (<43)* | 115 (89.84) | 53 (91.38) | 62 (88.57) |
| *High (43+)* | 13 (10.16) | 5 (8.62) | 8 (11.43) |
|  |  |  |  |

*Note: Absolute and relative frequencies are reported.*
